# Market impact shapes competitive advantage of investment strategies in financial markets

**DOI:** 10.1371/journal.pone.0260373

**Published:** 2022-02-03

**Authors:** Wen-Juan Xu, Li-Xin Zhong

**Affiliations:** 1 School of Law, Zhejiang University of Finance and Economics, Hangzhou, Zhejiang, China; 2 School of Finance and Coordinated Innovation Center of Wealth Management and Quantitative Investment, Zhejiang University of Finance and Economics, Hangzhou, Zhejiang, China; Education University of Hong Kong, CHINA

## Abstract

The formation of an efficient market depends on the competition between different investment strategies, which accelerates all available information into asset prices. By incorporating market impact and two kinds of investment strategies into an agent-based model, we have investigated the coevolutionary mechanism of different investment strategies and the role of market impact in shaping a competitive advantage in financial markets. The coevolution of history-dependent strategies and reference point strategies depends on the levels of market impact and risk tolerance. For low market impact and low risk tolerance, the majority-win effect makes the trend-following strategies become dominant strategies. For high market impact and low risk tolerance, the minority-win effect makes the trend-rejecting strategies coupled with trend-following strategies become dominant strategies. The coupled effects of price fluctuations and strategy distributions have been investigated in depth. A U-shape distribution of history-dependent strategies is beneficial for a stable price, which is destroyed by the existence of reference point strategies with low risk tolerance. A *δ*-like distribution of history-dependent strategies leads to a large price fluctuation, which is suppressed by the existence of reference point strategies with high risk tolerance. The strategies that earn more in an inefficient market lose more in an efficient market. Such a result gives us another explanation for the principle of risk-profit equilibrium in financial markets: high return in an inefficient market should be coupled with high risk in an efficient market, low return in an inefficient market should be coupled with low risk in an efficient market.

## Introduction

Complex behaviors like self-organization and phase transition are ubiquitous in social, economic, biological and physical systems [1–6]. A noted feature of these systems is that a variety of macroscopic changes usually appear spontaneously and unpredictably. In financial markets, whether the stock prices are predictable or not is usually taken as an indicator for market efficiency [7–10]. In an efficient market, the stock prices can fully reflect all available information. An advantageous condition would lead to an increase in the stock prices and a disadvantageous condition would lead to a decrease in the stock prices, which results from people’s competition for limited resources [11–15]. People’s investment strategy plays an important role in the competition.

In financial markets, people may adopt different kinds of strategies to make their investment [16–22]. An individual with a history-dependent strategy usually makes his buying and selling decision according to historical information, like the changing tendencies of stock prices and trading volumes [23–26]. An individual with a reference point strategy usually makes his buying and selling decisions according to his subjective evaluation of a given stock, which is called the reference point effect in behavioral finance [27–31]. The models employed in exploring the evolutionary dynamics of complex financial systems have so far been limited to the population with a typical kind of investment strategies [32–38]. The coupled effects of different kinds of investment strategies on the evolution of complex behaviors in financial systems, especially their competitive advantage under different market environments, are short of discussion in depth.

In this paper we examine the change of competitive advantage of investment strategies under the environments with different levels of market impact. Market impact is the effect that a buyer or a seller pushes the market to move toward the direction that he tried to refrain from [39–43]. In the stock market with low market impact, the information enters the transaction price with relatively long time delay. Therefore, the stock price is usually predictable. In the stock market with high market impact, the information enters the transaction price instantly. Therefore, the stock price is usually unpredictable. Depending upon the minority game, the effect of market impact on the evolution of stock prices has been investigated [44]. There exists a transition point, below which the stock price fluctuates greatly and above which the stock price is relatively stable. Depending upon the evolutionary minority game, the effect of market impact on the evolution of stock prices has been investigated [45]. The role of market impact in the evolution of stock prices is closely related to the existence of different kinds of investors. As the momentum traders exist, the market impact may be reduced or promoted.

Exploring the competitive advantage of investment strategies under different levels of market impact can tell us the following three questions: How are the stock prices affected by the coexistence of different kinds of investment strategies? Whether or not can the investment strategies always play their role in the evolution of stock prices independently? How does the market impact affect the competitive advantage of different investment strategies? The following are our main findings.

(1)The stock price fluctuations are closely related to the market impact and the ratio of individuals with history-dependent strategies and reference point strategies. For low market impact, the stock prices are relatively volatile and the existence of heterogeneous reference point strategies helps suppress the price fluctuations. For high market impact, the stock prices are relatively stable and the existence of homogeneous reference point strategies promotes price fluctuations.

(2)The coevolution of history-dependent strategies and reference point strategies is related to the levels of market impact and risk tolerance. For low market impact and low risk tolerance, the majority-win effect makes the trend-following strategies become dominant strategies. For high market impact and low risk tolerance, a heterogeneous distribution of history-dependent strategies is easy to be destroyed by homogeneous reference point strategies. The minority-win effect makes the trend-rejecting strategies coupled with trend-following strategies become dominant strategies. A heterogeneous distribution of reference point strategies is relatively stable, which is not easy to be affected by the existence of history-dependent strategies.

(3)The competitive advantage of investment strategies is closely related to the market impact. For low market impact, the strategy that promotes the price fluctuations outperforms the strategy that suppresses the price fluctuations. For high market impact, the strategy that suppresses the price fluctuations outperforms the strategy that promotes the price fluctuations. The effect of high risk and high return, low risk and low return is found.

The rest of the paper is organized as follows. The model with market impact and two kinds of investment strategies is introduced in section two. The simulation results are presented in section three. A theoretical analysis is given in section four. The conclusions are drawn in section five.

## The model

### Buying-selling actions and payoffs

The present model describes such a scenario where a large number *N* of individuals have to take one of the three actions at each time step: buying (a=+1), selling (a=-1) and taking no action (a = 0). The net payoff *P*_*i*_ of a buying-selling action to an individual *i* is

$$\begin{array}{c} P_{i}=P_{sell}-P_{buy}, \end{array}$$ (1)

in which *P*_*sell*_ and *P*_*buy*_ are the transaction prices at different times. There exists short sales mechanism in the present model. The amount of shares each individual holds are confined to *k*_*min*_ ≤ *k* ≤ *k*_*max*_. The individuals with minimum shares will not sell further and the individuals with maximum shares will not buy further.

The individuals with history-dependent strategies make their buying and selling decisions as follows. For an individual i with strategy *g*_*i*_, facing a typical history of price change, he follows the history prediction with probability *g*_*i*_ and rejects the history prediction with probability 1 − *g*_*i*_. The individuals with reference point strategies make their buying and selling decisions as follows. If the stock price is less than an individual’s expected price $p_{i}^{exp}$, he buys the stock with probability $\frac{p_{i}^{exp}-P\left(t\right)}{P(t)}$. If the stock price is greater than an individual’s expected price $p_{i}^{exp}$, he sells the stock with probability $\frac{P\left(t\right)-p_{i}^{exp}}{P(t)}$.

### Evolution of prices and available information

The price dynamics is related to the attendance buying and selling the stocks. More sellers than buyers leads to a decrease in stock prices. More buyers than sellers leads to an increase in stock prices. Following the work done in ref. [46], the return *R*(*t*) in trading is given by

$$\begin{array}{c} R\left(t\right)\equiv logP\left(t\right)-logP\left(t-1\right)=\frac{A(t)}{\lambda }, \end{array}$$ (2)

in which *A*(*t*) is the difference in the numbers of individuals buying and selling the stocks at time *t*, *A*(*t*) = *N*_*buy*_ − *N*_*sell*_, λ is called liquidity controlling price sensitivity on attendance. Therefore, the time-dependent price is

$$\begin{array}{c} P\left(t\right)=P\left(t-1\right)e^{\frac{N_{buy}-N_{sell}}{\lambda }}. \end{array}$$ (3)

Market impact is related to the discrepancy between an individual’s expected price and the actual transaction price [47, 48], which depends on how long the individual queues up for his transaction turn. During such a sequential process of clearing the deals, the transaction price ranges from its present value to a new value, which is satisfied with the equation [47]

$$\begin{array}{c} P^{tr}\left(t\right)=\left(1-\beta \right)P\left(t-1\right)+\beta P\left(t\right). \end{array}$$ (4)

For *β* = 0, the buyers and the sellers finish their transactions with the latest historical price *P*(*t* − 1). For *β* = 1, the buyers and the sellers finish their transactions with the instant price *P*(*t*).

There is a time series of latest historical prices, which is *m*-bit long and can be depended upon as available information for an individual to make his buying and selling decisions. For an individual with a history-dependent investment strategy, he follows the history-dependent trend prediction with probability *g* and rejects it with probability 1 − *g*. For example, facing a price history ↑↑↑, if the history-dependent trend prediction is ↑, an individual *i* makes a buying decision with probability *g*_*i*_ and makes a selling decision with probability 1 − *g*_*i*_. If the history-dependent trend prediction is ↓, he makes a selling decision with probability *g*_*i*_ and makes a buying decision with probability 1 − *g*_*i*_. Therefore, *g*_*i*_ is a history-dependent strategy of individual *i*.

For an individual with a reference point investment strategy, he does not follow the history-dependent trend prediction. The *m*′-bit long latest historical prices are only averaged as a benchmark for an individual to determine his reference point. For example, facing an average price $\bar{P}$, if an individual *i*’s reference point $p_{i}^{ref}$ is within the range of $\bar{P}e^{-\alpha G_{i}}\leq p_{i}^{ref}\leq \bar{P}e^{\alpha G_{i}}$, in which *G*_*i*_ represents individual *i*’s risk tolerance and *α* is a constant, he keeps his reference point. Or else, he will randomly choose a new reference point within the range of $\bar{P}e^{-\alpha G_{i}}\leq p_{i}^{ref}\leq \bar{P}e^{\alpha G_{i}}$. Similar to that in ref. [49], the risk tolerance is incorporated into the present model as follows. Initially, each individual randomly chooses his own *G*_*i*_ within the range of *G*_*i*_ ∈ [*G*_*min*_, *G*_*max*_]. If *G*_*min*_ = *G*_*max*_, all the individuals have the same risk tolerance. For an individual i with a large *G*_*i*_, his reference point strategy $p_{i}^{ref}$ is relatively stable. For an individual i with a small *G*_*i*_, he is quite possible update his reference point strategy $p_{i}^{ref}$ frequently.

### Heterogeneous beliefs and evolution of strategies

Similar to the strategies in the evolutionary minority game [50], the history-dependent investment strategy is related to the latest m-bit long stock prices. For example, for *m* = 3, the m-bit-string (xyz) and the latest historical outcome *w* are (↓↓↓) ↑, (↓↓↑) ↓, (↓↑↓) ↓, (↓↑↑) ↑, (↑↓↓) ↓, (↑↓↑) ↑, (↑↑↓) ↓, (↑↑↑) ↑, in which ↑ represents the rise of price and ↓ represents the drop of price. Faced with the latest change of stock prices (↓↓↓), an individual might simply predict the same outcome ↑ as that registered in the memory. The individual will hence make a buying decision. An individual is also possible to predict the outcome ↓ contradictory to that registered in the memory. The individual will hence make a selling decision. Similar to the work done in ref. [50], each individual is assigned a strategy *g*. Following a given m-bit long price change, the individual will follow the current prediction with probability *g* and reject the current prediction with probability 1 − *g*.

The history-dependent strategy *g* is within the range of 0 ≤ *g* ≤ 1. For a homogeneous population, they have similar strategies and the strategy distribution is narrow. For a heterogeneous population, they have different strategies and the strategy distribution is wide. The evolution of *g* depends upon the score of the strategy. Similar to the work done in the Minority Game [19, 51], the strategy score is related to the attainment of the individual using this strategy. If the individual adopting this strategy gets more, his strategy score increases and he would keep his strategy. If the individual adopting this strategy loses more, his strategy score decreases and he would update his strategy. The strategy score can be got according to the following equation

$$\begin{array}{c} S_{g}=\Sigma_{\Delta n}\left(P^{sell}-P^{buy}\right), \end{array}$$ (5)

in which Δ*n* is the times that individual i uses strategy *g*. If *S*_*g*_ is less than a threshold *S*_*th*_, a new strategy *g*′ is randomly chosen within the range of $g-\frac{\varepsilon }{2}\leq g^{\prime }\leq g+\frac{\varepsilon }{2}$, in which *ε* is a pregiven small constant, and *S*_*g*_ is reset to *S*_*g*_ = 0.

In real-life considerations, valuing a company is usually subjective. According to the prospect theory, some kind of historical stock price might become an anchor for an individual to value a company [27], such as the highest price in a period of time [30]. In order to modelling the anchoring-and-conservative-adjustment estimation method [52], following the suggestions proposed by professor Yi-Cheng Zhang [49], we introduce a new investment strategy, called reference point strategy, into the present model.

The reference point strategy *p*^*ref*^ is established as an anchor for an individual to value a stock. *p*^*ref*^ is within the range of *p*^*ref*^ ≥ 0. For a homogeneous population, they have similar reference points and the distribution of reference points is narrow. For a heterogeneous population, they have different reference points and the distribution of reference points is wide. The evolution of *p*^*ref*^ depends upon the distance between an individual i’s reference point $p_{i}^{ref}$ and the average price $\bar{P}$ in the latest *m*′ steps. On condition that $p_{i}^{ref}<\bar{P}e^{-\alpha G_{i}}$ or $p_{i}^{ref}>\bar{P}e^{\alpha G_{i}}$, individual *i* updates his reference point.

### Predictability of stock prices

The predictability of stock prices is often used to measure the characteristics of price changes as some typical history occurs. Given the conditions that the history *χ* occurs with probability *ρ*(*χ*) and the mean change of prices is 〈Δ*P*|*χ*〉, the predictability *H* can be obtained [47]

$$\begin{array}{c} H=\sum_{\chi }\rho \left(\chi \right){\left\langle \Delta P|\chi \right\rangle }^{2}. \end{array}$$ (6)

For example, facing the changes in the historical prices ↑↑↑, if there are more ↑ than ↓ or more ↓ than ↑ following such a history, the price changes are predictable. The value of H should be large.

### Each individual’s accumulated wealth

Similar to the wealth in ref. [47], in the present model, each individual’s wealth is accumulated from his buying and selling actions. For an individual i, if his initial cash is *C*_*i*_(*t* = 0) = 0, his accumulated cash is

$$\begin{array}{c} C_{i}\left(T\right)=\Sigma_{t=0}^{T}\left(P^{sell}-P^{buy}\right), \end{array}$$ (7)

and the value of the stock in his hand is

$$\begin{array}{c} V_{i}^{stock}=k_{i}P^{tr}, \end{array}$$ (8)

in which *k*_*i*_ is the number of shares in his hand. Therefore, individual i’s accumulated wealth at time *T* is

$$\begin{array}{c} W_{i}=C_{i}\left(T\right)+V_{i}^{stock}=\Sigma_{t=0}^{T}\left(P^{sell}-P^{buy}\right)+k_{i}P^{tr}. \end{array}$$ (9)

The average wealth of the population is

$$\begin{array}{c} \bar{W}=\frac{\Sigma_{i=1}^{N}W_{i}}{N}. \end{array}$$ (10)

## Simulation results and discussions

### Reproduction of the stylized facts in real financial markets

Firstly, we examine whether the present model can reproduce the stylized facts found in real financial markets [53–62].

In real financial market, the distribution of absolute price returns shows a fat tail, which is satisfied with an equation *y* = *ax*^−*b*^ [63]. In the present model, by adjusting the ratio of the individuals with reference point strategies, we can reproduce the fat tail behavior, which means, by adjusting *ξ*, the power-law exponent *b* can range from a quite large *b* ∼ 9 to a small *b* ∼ 3.

**Fig 1:**
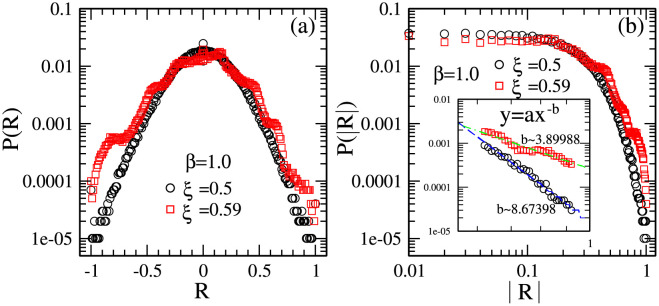
(a) The distributions of normalized price returns for the ratio of individuals with reference point strategies *ξ* = 0.5 (circles), 0.59 (squares); (b) the distributions of absolute normalized price returns for *ξ* = 0.5 (circles), 0.59 (squares); the inset in (b) is partial enlarged view within the range of ∣*R*∣∈[0.62, 0.92]. Other parameters are: the population size *N* = 5001, the memory size *m* = 3 and *m*′ = 10, the market impact *β* = 1, the maximal risk tolerance of reference point strategies *G*_*max*_ = 1000, the updating threshold of history-dependent strategies *S*_*th*_ = 0, the maximum drift of history-dependent strategies *ε* = 0.1, the liquidity $\lambda =\frac{N}{10}$, the constant $\alpha =\frac{10}{N}$, minimum and maximum shares holding *k*_*min*_ = -1, *k*_*max*_ = 1. Final data are obtained by averaging over 50 runs and 10^4^ times after 10^5^ relaxation times in each run. The initial conditions are: an individual’s risk tolerance *G*_*i*_ is uniformly distributed within the range of *G*_*i*_ ∈ [0, *G*_*max*_], an individual’s initial reference point $p_{i}^{ref}$ is uniformly distributed within the range of $p_{i}^{ref}\in \left[\bar{P}e^{-\alpha G_{i}},\bar{P}e^{\alpha G_{i}}\right]$, initial history-dependent strategy *g* is distributed uniformly within the range of *g* ∈ [0, 1], starting price *P*(*t* = 0) is randomly chosen within the range of *P* ∈ [0, 20], each individual’s initial number of shares *k* = 0, each individual’s initial wealth *W*_*i*_(*t* = 0) = 0, each individual’s initial cash *C*_*i*_(*t* = 0) = 0.

Fig 1(a) and 1(b) show the distributions of price returns and absolute price returns for different ratio of individuals with reference point strategies. For *ξ* = 0.5, which corresponds to the situation where half of the individuals adopt history-dependent strategies and another half of the individuals adopt reference point strategies, the distribution of price returns is similar to a Normal distribution. For *ξ* = 0.59, which corresponds to the situation where more than half of the individuals adopt reference point strategies, the distribution of price returns is similar to a fat-tail distribution found in real financial market. As we make a fit line to the simulation data in Fig 1(b), for *ξ* = 0.59, a power-law tail with exponent *b* ∼ 3.9 is found, for which the confidence interval is *b* ∈ [3.41, 4.39].

Such results indicate that, by adjusting the ratio of individuals with reference point strategies, the present model would effectively reproduce the stylized fact of a power-law distribution of price returns with exponent *b* ∼ 3 similar to that in refs. [44, 56, 57, 64–67]. It suggests that the fat-tail distribution of price returns in real financial markets might result from the existence of individuals with reference point strategies.

The Hurst exponent is usually used to identify the long memory in the time-dependent parameters [56, 68]. From the range of the exponent, we can identify whether the time-dependent parameter is random or long-range correlation. If the exponent is near *h* ∼ 0.5, the parameter is random. If the exponent is less than 0.5, it is noise. If the exponent is greater than 0.5, it is long-range correlation. Some empirical studies show that the time-dependent stock prices are long-range correlation [69, 70].

The detrended fluctuation analysis (DFA) method is usually used to measure the hurst exponent [71–73]. Given the time-dependent stock prices, *P*(*t*), t = 1, 2, 3, …, T, we can get the integrated series *y*(*l*) according to the equation $y\left(l\right)=\Sigma_{i=1}^{l}\left[P\left(i\right)-\bar{P}\right]$, in which *P*(*i*) and $\bar{P}$ are the *ith* and the average values respectively. Given the box length *S*, we can divide the integrated series into n boxes and get the detrended series *y*(*l*) − *y*_*S*_(*l*), in which *y*_*S*_(*l*) is the local trend in each box. The root-mean-square of *y*(*l*) can be quantified according to the equation $F\left(S\right)=\sqrt{\frac{\Sigma_{l=1}^{S}{\left[y\left(l\right)-y_{S}\left(l\right)\right]}^{2}}{S}}$. The Hurst exponent h can be got, *F*(*S*) ∼ *S*^−*h*^.

**Fig 2:**
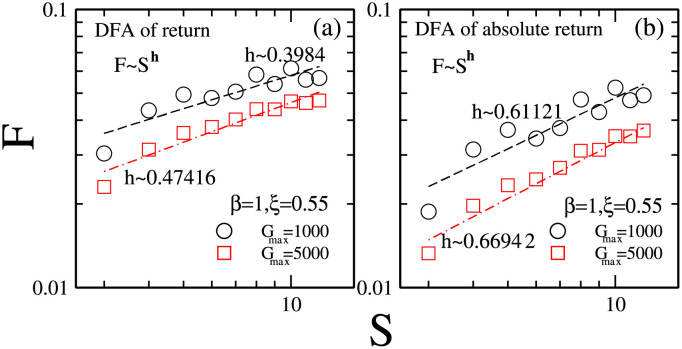
(a) The hurst exponent of price returns for the maximal risk tolerance of reference point strategies *G*_*max*_ = 1000 (circles), 5000 (squares); (b) the hurst exponent of absolute price returns for *G*_*max*_ = 1000 (circles), 5000 (squares). Other parameters are: the population size *N* = 5001, the memory size *m* = 3 and *m*′ = 10, the market impact *β* = 1, the ratio of individuals with reference point strategies *ξ* = 0.55, the updating threshold of history-dependent strategies *S*_*th*_ = 0, the maximum drift of history-dependent strategies *ε* = 0.1, the liquidity $\lambda =\frac{N}{10}$, the constant $\alpha =\frac{10}{N}$, minimum and maximum shares holding *k*_*min*_ = -1, *k*_*max*_ = 1. Final data are obtained by averaging over 50 runs and 10^4^ times after 10^5^ relaxation times in each run. The initial conditions are: an individual’s risk tolerance *G*_*i*_ is uniformly distributed within the range of *G*_*i*_ ∈ [0, *G*_*max*_], an individual’s initial reference point $p_{i}^{ref}$ is uniformly distributed within the range of $p_{i}^{ref}\in \left[\bar{P}e^{-\alpha G_{i}},\bar{P}e^{\alpha G_{i}}\right]$, initial history-dependent strategy *g* is distributed uniformly within the range of *g* ∈ [0, 1], starting price *P*(*t* = 0) is randomly chosen within the range of *P* ∈ [0, 20], each individual’s initial number of shares *k* = 0, each individual’s initial wealth *W*_*i*_(*t* = 0) = 0, each individual’s initial cash *C*_*i*_(*t* = 0) = 0.

Fig 2(a) shows the hurst exponent of price returns for different maximal risk tolerance of individuals with reference point strategies. For *G*_*max*_ = 1000, which corresponds to the situation where the distribution of reference points is relatively narrow, the hurst exponent of price returns is *h* ∼ 0.4, for which the confidence interval is *h* ∈ [0.23, 0.56]. For *G*_*max*_ = 5000, which corresponds to the situation where the distribution of reference points is relatively broader, the hurst exponent of price returns is *h* ∼ 0.47, for which the confidence interval is *h* ∈ [0.36, 0.59].

In Fig 2(b) we further plot the hurst exponent of absolute price returns. As we make a fit line to the simulation data, for *G*_*max*_ = 1000, the hurst exponent is *h* ∼ 0.61, for which the confidence interval is *h* ∈ [0.40, 0.82]. For *G*_*max*_ = 5000, the hurst exponent is *h* ∼ 0.67, for which the confidence interval is *h* ∈ [0.54, 0.80].

Such results indicate that, by adjusting the maximal risk tolerance of individuals with reference point strategies, the present model would effectively reproduce the stylized fact of the hurst exponent of absolute price returns *h* ∼ 0.7. It suggests that the long-range correlation of price returns in real financial markets might result from the existence of the individuals with reference point strategies.

### Coupled effects of market impact and coevolution of investment strategies on price movement

**Fig 3:**
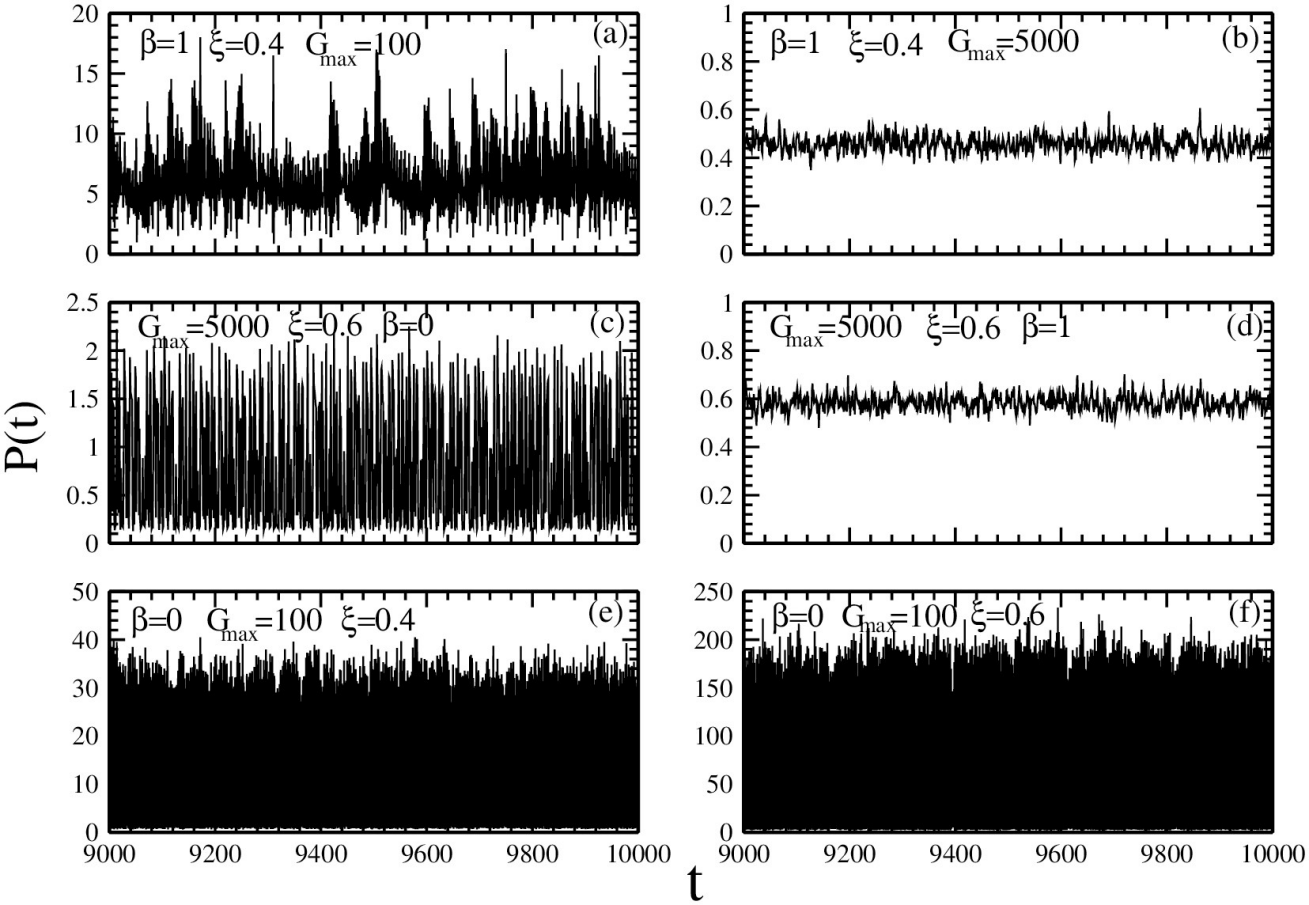
The dynamic stock prices *P* for different combinations of market impact *β*, maximal risk tolerance *G*_*max*_ of reference point strategies, ratio *ξ* of individuals with reference point strategies. (a) *β* = 1, *ξ* = 0.4 and *G*_*max*_ = 100; (b) *β* = 1, *ξ* = 0.4 and *G*_*max*_ = 5000; (c) *G*_*max*_ = 5000, *ξ* = 0.6 and *β* = 0; (d)*G*_*max*_ = 5000, *ξ* = 0.6 and *β* = 1; (e) *β* = 0, *G*_*max*_ = 100 and *ξ* = 0.4; (f) *β* = 0, *G*_*max*_ = 100 and *ξ* = 0.6. Other parameters are: the population size *N* = 5001, the memory size *m* = 3 and *m*′ = 10, the updating threshold of history-dependent strategies *S*_*th*_ = 0, the maximum drift of history-dependent strategies *ε* = 0.1, the liquidity $\lambda =\frac{N}{10}$, the constant $\alpha =\frac{10}{N}$, minimum and maximum shares holding *k*_*min*_ = -1, *k*_*max*_ = 1. The initial conditions are: an individual’s risk tolerance *G*_*i*_ is uniformly distributed within the range of *G*_*i*_ ∈ [0, *G*_*max*_], an individual’s initial reference point $p_{i}^{ref}$ is uniformly distributed within the range of $p_{i}^{ref}\in \left[\bar{P}e^{-\alpha G_{i}},\bar{P}e^{\alpha G_{i}}\right]$, initial history-dependent strategy *g* is distributed uniformly within the range of *g* ∈ [0, 1], starting price *P*(*t* = 0) is randomly chosen within the range of *P* ∈ [0, 20], each individual’s initial number of shares *k* = 0, each individual’s initial wealth *W*_*i*_(*t* = 0) = 0, each individual’s initial cash *C*_*i*_(*t* = 0) = 0.

Firstly, we examine how the market impact and the investment strategies affect the evolution of stock prices. Fig 3 presents the dynamic stock prices for different combinations of market impact and investment strategies.

Fig 3(a) and 3(b) show that, for high market impact *β* = 1, the fluctuation of stock prices is determined by the maximal risk tolerance *G*_*max*_. As the maximal risk tolerance is relatively small, *G*_*max*_ = 100, there is a large price fluctuation. As the maximal risk tolerance is relatively large, *G*_*max*_ = 5000, there is a small price fluctuation.

Fig 3(c) and 3(d) show that, as the maximal risk tolerance of the individuals with reference point strategies is large, *G*_*max*_ = 5000, the price fluctuation is determined by the market impact. As the market impact is small, *β* = 0, there is a large price fluctuation. As the market impact is large, *β* = 1, there is a small price fluctuation.

Fig 3(e) and 3(f) show that, as both the market impact and the maximal risk tolerance of the individuals with reference point strategies are small, i.e. *β* = 0 and *G*_*max*_ = 100, the price fluctuation is quite large. The change in the ratio of the individuals with history-dependent strategies and reference point strategies only has little effect on the change of price fluctuation.

Such results indicate that the price fluctuation is determined by the coupling of different investment strategies. Within the range where the price is relatively stable, *β* = 1 or *G*_*max*_ = 5000, the existence of another investment strategy is quite possible to destroy such a stable state. We can understand such results as follows. The change in stock prices is determined by the difference in the numbers of individuals buying and selling the stocks. For *β* = 1 and *G*_*max*_ = 5000, the heterogeneous population lead to a small value of the difference in the numbers of individuals buying and selling the stocks. Therefore, the price is somewhat stable. For *β* = 0 and *G*_*max*_ = 100, the homogeneous population lead to a large value of the difference in the numbers of individuals buying and selling the stocks. Therefore, the price fluctuation is quite large.

**Fig 4:**
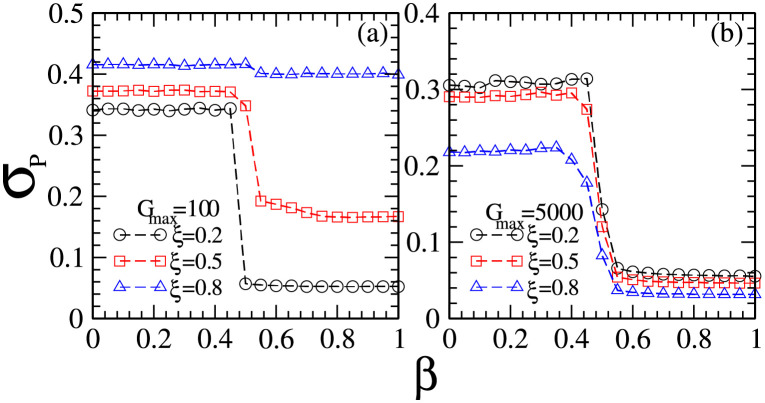
The standard deviation *σ*_*P*_ of normalized stock prices as a function of market impact *β* for different combinations of maximal risk tolerance *G*_*max*_ of reference point strategies and the ratio *ξ* of individuals with reference point strategies. (a) *G*_*max*_ = 100 and *ξ* = 0.2 (circles), 0.5 (squares), 0.8 (triangles); (b) *G*_*max*_ = 5000 and *ξ* = 0.2 (circles), 0.5 (squares), 0.8 (triangles). Other parameters are: the population size *N* = 5001, the memory size *m* = 3 and *m*′ = 10, the updating threshold of history-dependent strategies *S*_*th*_ = 0, the maximum drift of history-dependent strategies *ε* = 0.1, the liquidity $\lambda =\frac{N}{10}$, the constant $\alpha =\frac{10}{N}$, minimum and maximum shares holding *k*_*min*_ = -1, *k*_*max*_ = 1. Final data are obtained by averaging over 200 runs and 10^4^ times after 10^5^ relaxation times in each run. The initial conditions are: an individual’s risk tolerance *G*_*i*_ is uniformly distributed within the range of *G*_*i*_ ∈ [0, *G*_*max*_], an individual’s initial reference point $p_{i}^{ref}$ is uniformly distributed within the range of $p_{i}^{ref}\in \left[\bar{P}e^{-\alpha G_{i}},\bar{P}e^{\alpha G_{i}}\right]$, initial history-dependent strategy *g* is distributed uniformly within the range of *g* ∈ [0, 1], starting price *P*(*t* = 0) is randomly chosen within the range of *P* ∈ [0, 20], each individual’s initial number of shares *k* = 0, each individual’s initial wealth *W*_*i*_(*t* = 0) = 0, each individual’s initial cash *C*_*i*_(*t* = 0) = 0.

In order to get a clear view on the relationship between the price fluctuations and the coupling of market impact and investment strategies, in Fig 4(a) and 4(b) we plot the standard deviation *σ*_*P*_ of normalized stock prices as a function of market impact *β* for different combinations of maximal risk tolerance *G*_*max*_ and the ratio *ξ* of individuals with reference point strategies. The normalize *σ*_*P*_ is calculated from the normalized stock price, $P^{\prime }=\frac{P-P_{min}}{P_{max}-P_{min}}$, in which *P*_*max*_ and *P*_*min*_ are the maximal and minimal stock prices respectively within the time window.

From Fig 4(a) and 4(b) we find that the changing tendency of *σ*_*P*_ vs *β* is independent of *G*_*max*_ and *ξ*. There exists a transition point *β* = 0.5, within the range of 0 ≤ *β* < 0.5, *σ*_*P*_ keeps a relatively high value. Within the range of 0.5 < *β* ≤ 1, *σ*_*P*_ keeps a relatively low value. Such results indicate that a higher level of market impact suppresses the price fluctuations.

Comparing the role of *ξ* in the price fluctuation in Fig 4(a) and 4(b), we find that an increase in the ratio of individuals with reference point strategies has contradictory effects on the price fluctuations. As the maximal risk tolerance of reference point strategies is relatively low, *G*_*max*_ = 100, which corresponds to the situation where the distribution of reference points is relatively narrow, an increase in *ξ* leads to an overall increase in *σ*_*P*_. As the maximal risk tolerance of reference point strategies is relatively high, *G*_*max*_ = 5000, which corresponds to the situation where the distribution of reference points is relatively wide, an increase in *ξ* leads to an overall decrease in *σ*_*P*_.

Such results indicate that a low level of risk tolerance promotes price fluctuations and a high level of risk tolerance suppresses price fluctuations. We can understand such results as follows. As most of the people have low risk tolerance, they are quite possible to make a trade frequently. Therefore, the stock price fluctuates greatly. As most of the people have high risk tolerance, they are quite possible to take a hold for a long time. Therefore, the stock price changes little.

**Fig 5:**
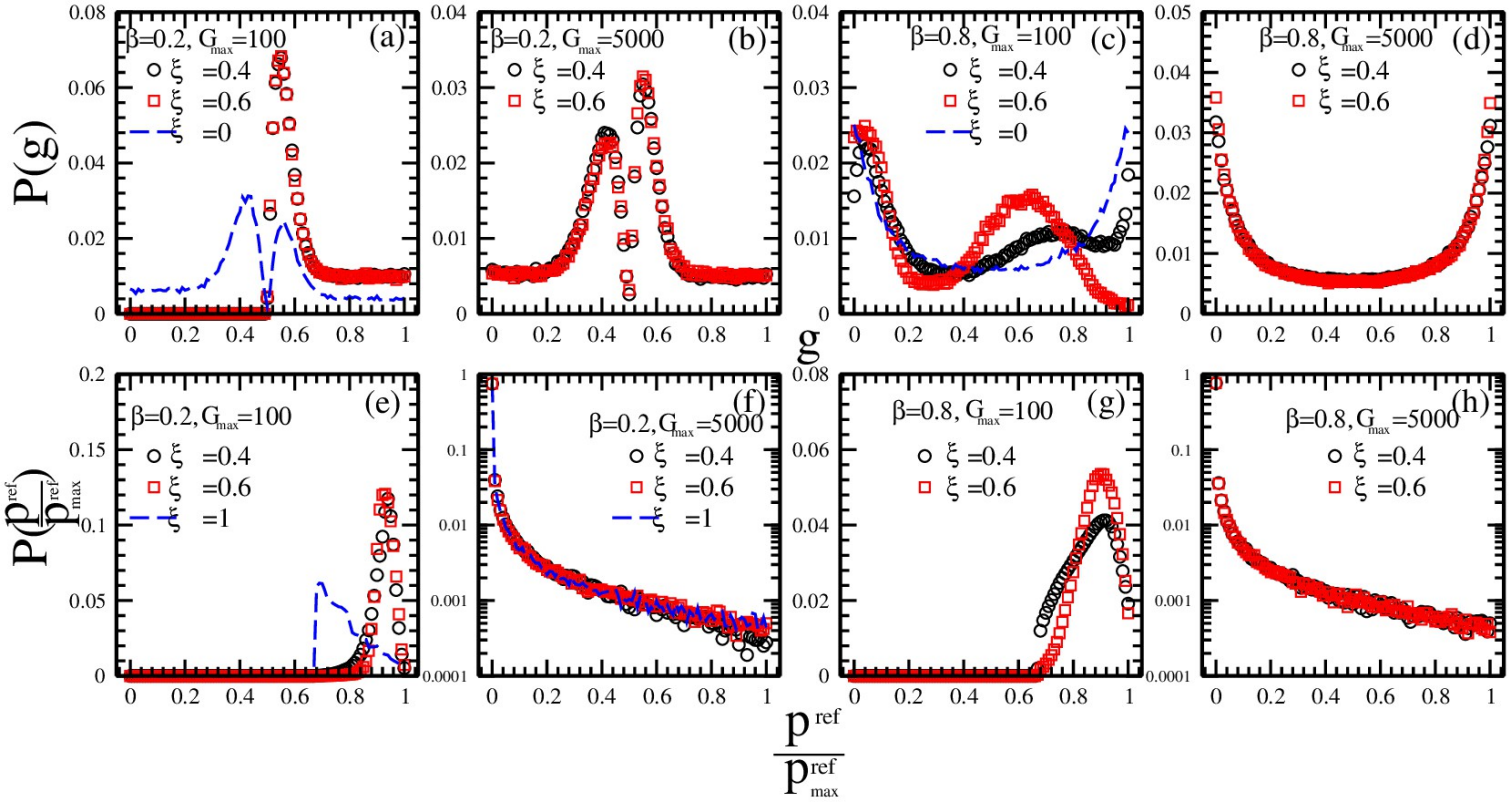
The distributions of history-dependent strategies (a, b, c, d) and reference point strategies (e, f, g, h) for different combinations of market impact *β*, maximal risk tolerance *G*_*max*_ of reference point strategies and ratio *ξ* of individuals with reference point strategies. (a) and (e) *β* = 0.2, *G*_*max*_ = 100 and *ξ* = 0.4 (circles), 0.6 (squares); (b) and (f) *β* = 0.2, *G*_*max*_ = 5000 and *ξ* = 0.4 (circles), 0.6 (squares); (c) and (g) *β* = 0.8, *G*_*max*_ = 100 and *ξ* = 0.4 (circles), 0.6 (squares); (d) and (h) *β* = 0.8, *G*_*max*_ = 5000 and *ξ* = 0.4 (circles), 0.6 (squares). Other parameters are: the population size *N* = 5001, the memory size *m* = 3 and *m*′ = 10, the updating threshold of history-dependent strategies *S*_*th*_ = 0, the maximum drift of history-dependent strategies *ε* = 0.1, the liquidity $\lambda =\frac{N}{10}$, the constant $\alpha =\frac{10}{N}$, minimum and maximum shares holding *k*_*min*_ = -1, *k*_*max*_ = 1. The initial conditions are: an individual’s risk tolerance *G*_*i*_ is uniformly distributed within the range of *G*_*i*_ ∈ [0, *G*_*max*_], an individual’s initial reference point $p_{i}^{ref}$ is uniformly distributed within the range of $p_{i}^{ref}\in \left[\bar{P}e^{-\alpha G_{i}},\bar{P}e^{\alpha G_{i}}\right]$, initial history-dependent strategy *g* is distributed uniformly within the range of *g* ∈ [0, 1], starting price *P*(*t* = 0) is randomly chosen within the range of *P* ∈ [0, 20], each individual’s initial number of shares *k* = 0, each individual’s initial wealth *W*_*i*_(*t* = 0) = 0, each individual’s initial cash *C*_*i*_(*t* = 0) = 0.

In order to examine how different investment strategies affect each other, in Fig 5 we plot the distributions of history-dependent strategies and reference point strategies for different combinations of market impact *β*, maximal risk tolerance *G*_*max*_ of reference point strategies and ratio *ξ* of individuals with reference point strategies.

Comparing the results in Fig 5(a) and 5(c) with the results in Fig 5(b) and 5(d), we find that the existence of the reference point strategies with low risk tolerance can effectively affect the distributions of history-dependent strategies while the existence of the reference point strategies with high risk tolerance has little effect on the change of the distributions of history-dependent strategies. Within the range where the market impact is low, for *ξ* = 0, which corresponds to the situation where all the individuals adopt history-dependent strategies, the history-dependent strategies cluster around *g* < 0.5 or *g* > 0.5 (slash lines in Fig 5(a)). Because of the existence of the reference point strategies with low risk tolerance, only the trend-following strategies become dominant strategy. Within the range where the market impact is high, for *ξ* = 0, the history-dependent strategies have a U-shape distribution clustering around *g* ∼ 0 and *g* ∼ 1 (slash lines in Fig 5(c)). Because of the existence of the reference point strategies with low risk tolerance, only the trend-rejecting strategies become dominant strategy.

Such results can be understood as follows. As the market impact is low, the majority-win effect would make the trend-following or trend-rejecting investment become dominant. The existence of the individuals with reference point strategies is quite possible to lead to an increase in the number of individuals with trend-following behavior, which would finally make the trend-following investment become dominant behavior. As the market impact is high, the minority-win effect would make the trend-following investment coupled with trend-rejecting investment become dominant. The existence of the individuals with reference point strategies is quite possible to lead to an increase in the number of individuals with trend-following behavior, which would finally make the trend-rejecting investment become dominant behavior.

Comparing the results in Fig 5(e) and 5(g) with the results in Fig 5(f) and 5(h), we find that whether or not the existence of history-dependent strategies can affect the distribution of reference point strategies is not related to the market impact but depends on the maximum risk tolerance *G*_*max*_ of the individuals with reference point strategies. As the maximum risk tolerance is low, for *ξ* = 1, which corresponds to the situation where all the individuals adopt reference point strategies, the reference point strategies present a narrow distribution clustering around $\frac{p^{ref}}{p_{max}^{ref}}∼0.65$ (slash lines in Fig 5(e)). An increase in the number of individuals with history-dependent strategies leads to an increase in the dominant strategy, $\frac{p^{ref}}{p_{max}^{ref}}∼0.9$. As the maximum risk tolerance is high, for *ξ* = 1, the reference point strategies present a broad distribution clustering around $\frac{p^{ref}}{p_{max}^{ref}}∼0$ (slash lines in Fig 5(f)). The existence of history-dependent strategies has little effect on the change of the distribution of reference point strategies.

Such results can be understood as follows. As the risk tolerance is low, the change in the stock prices is quite possible to make the individuals with reference point strategies update their strategy frequently. The existence of the individuals with history-dependent strategies make the stock prices change continuously, which would finally make the reference point strategies cluster around the average value of stock prices. As the risk tolerance is high, i.e. *G*_*i*_ = 5000, because the initial strategies scatter within a broader range of $p_{i}^{ref}\in \left[\bar{P}e^{-10},\bar{P}e^{10}\right]$, only the individuals far away from $\bar{P}$ is possible to update their strategies. Therefore, after an initial updating of individual strategies, the reference point strategies keep relatively stable and the strategies near the average price become dominant.

**Fig 6:**
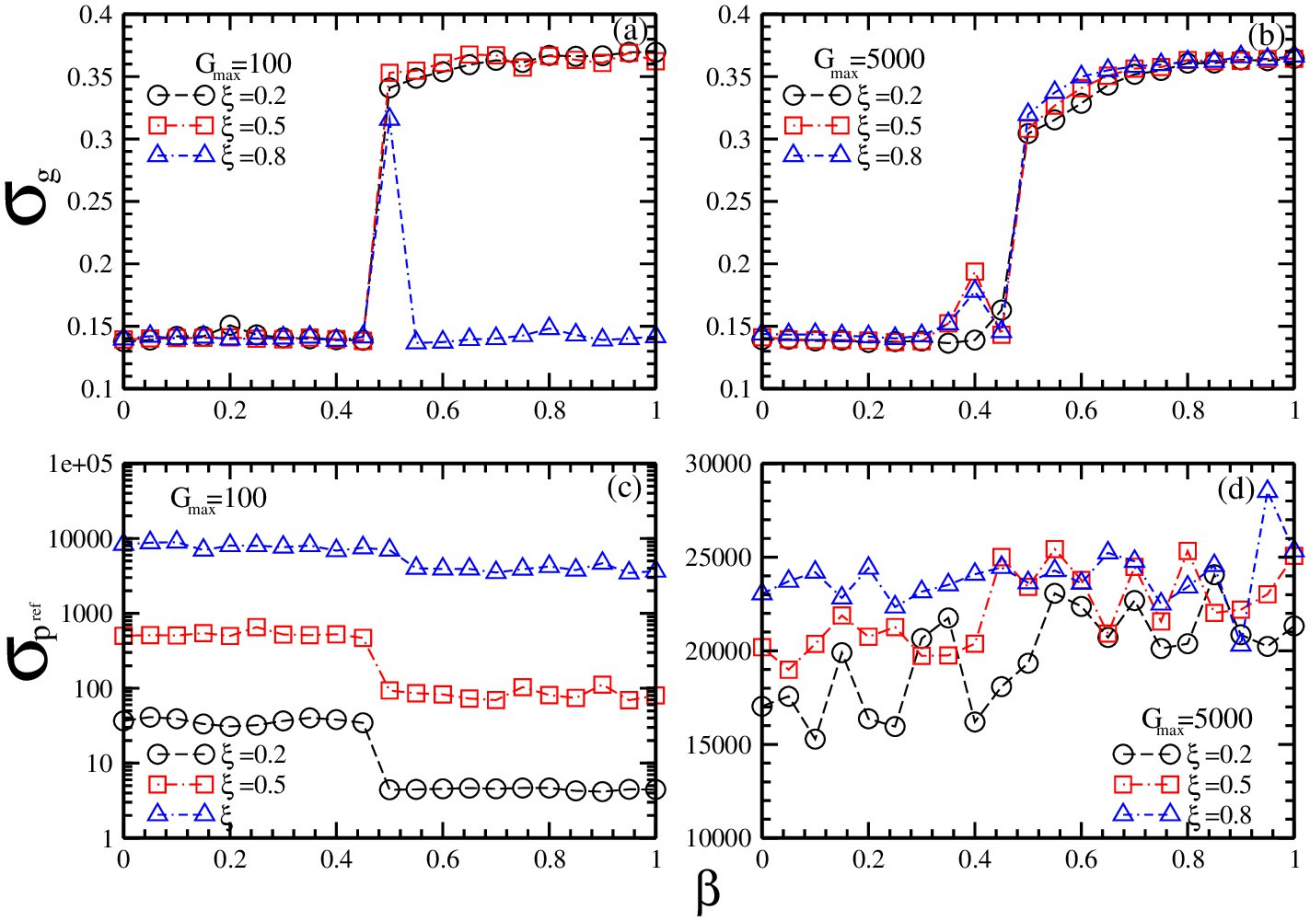
The standard deviation of investment strategies as a function of market impact *β* for different combinations of maximal risk tolerance *G*_*max*_ of reference point strategies and the ratio *ξ* of individuals with reference point strategies. (a) *σ*_*g*_ for *G*_*max*_ = 100 and *ξ* = 0.2 (circles), 0.5 (squares), 0.8 (triangles); (b) *σ*_*g*_ for *G*_*max*_ = 5000 and *ξ* = 0.2 (circles), 0.5 (squares), 0.8 (triangles); (c) $\sigma_{p^{ref}}$ for *G*_*max*_ = 100 and *ξ* = 0.2 (circles), 0.5 (squares), 0.8 (triangles); (d) $\sigma_{p^{ref}}$ for *G*_*max*_ = 5000 and *ξ* = 0.2 (circles), 0.5 (squares), 0.8 (triangles). Other parameters are: the population size *N* = 5001, the memory size *m* = 3 and *m*′ = 10, the updating threshold of history-dependent strategies *S*_*th*_ = 0, the maximum drift of history-dependent strategies *ε* = 0.1, the liquidity $\lambda =\frac{N}{10}$, the constant $\alpha =\frac{10}{N}$, minimum and maximum shares holding *k*_*min*_ = -1, *k*_*max*_ = 1. The initial conditions are: an individual’s risk tolerance *G*_*i*_ is uniformly distributed within the range of *G*_*i*_ ∈ [0, *G*_*max*_], an individual’s initial reference point $p_{i}^{ref}$ is uniformly distributed within the range of $p_{i}^{ref}\in \left[\bar{P}e^{-\alpha G_{i}},\bar{P}e^{\alpha G_{i}}\right]$, initial history-dependent strategy *g* is distributed uniformly within the range of *g* ∈ [0, 1], starting price *P*(*t* = 0) is randomly chosen within the range of *P* ∈ [0, 20], each individual’s initial number of shares *k* = 0, each individual’s initial wealth *W*_*i*_(*t* = 0) = 0, each individual’s initial cash *C*_*i*_(*t* = 0) = 0.

In order to get a clear view on the exact conditions for the coevolution of history-dependent strategies and reference point strategies, in Fig 6 we plot the standard deviation of investment strategies as a function of market impact *β* for different maximal risk tolerance *G*_*max*_ of reference point strategies and different ratio *ξ* of individuals with reference point strategies.

Fig 6(a) and 6(b) show that the history-dependent strategies are only affected by the reference point strategies with low risk tolerance. For high market impact, as the ratio of individuals with reference point strategies is larger than the ratio of individuals with history-dependent strategies, an increase in *ξ* leads to a decrease in *σ*_*g*_.

Fig 6(c) and 6(d) show that the standard deviation $\sigma_{p^{ref}}$ of reference point strategies is closely related to the ratio of the individuals with reference point strategies. As the ratio of individuals with reference point strategies increases, the standard deviation of reference point strategies increases.

Comparing the results in Fig 6 with the results in Fig 4, we find that, for low risk tolerance, an increase in $\sigma_{p^{ref}}$ is related to an increase in *σ*_*P*_. Such a result indicates that an increase in $\sigma_{p^{ref}}$ with low risk tolerance should result from the change of stock prices. For high risk tolerance, an increase in $\sigma_{p^{ref}}$ is related to a decrease in *σ*_*P*_. Such a result indicates that an increase in $\sigma_{p^{ref}}$ with high risk tolerance should result from an increase in the ratio of individuals with reference point strategies.

**Fig 7:**
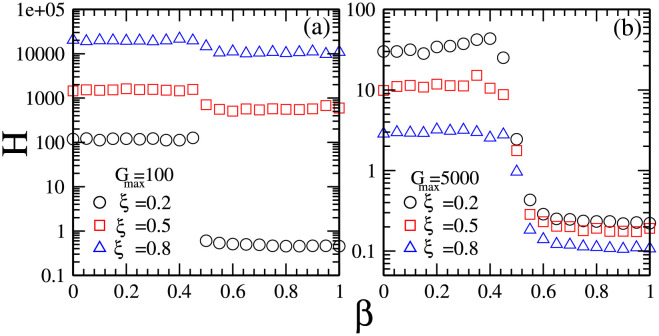
The predictability *H* of stock prices as a function of market impact *β* for different combinations of maximal risk tolerance *G*_*max*_ of reference point strategies and the ratio *ξ* of individuals with reference point strategies. (a) *G*_*max*_ = 100 and *ξ* = 0.2 (circles), 0.5 (squares), 0.8 (triangles); (b) *G*_*max*_ = 5000 and *ξ* = 0.2 (circles), 0.5 (squares), 0.8 (triangles). Other parameters are: the population size *N* = 5001, the memory size *m* = 3 and *m*′ = 10, the updating threshold of history-dependent strategies *S*_*th*_ = 0, the maximum drift of history-dependent strategies *ε* = 0.1, the liquidity $\lambda =\frac{N}{10}$, the constant $\alpha =\frac{10}{N}$, minimum and maximum shares holding *k*_*min*_ = -1, *k*_*max*_ = 1. The initial conditions are: an individual’s risk tolerance *G*_*i*_ is uniformly distributed within the range of *G*_*i*_ ∈ [0, *G*_*max*_], an individual’s initial reference point $p_{i}^{ref}$ is uniformly distributed within the range of $p_{i}^{ref}\in \left[\bar{P}e^{-\alpha G_{i}},\bar{P}e^{\alpha G_{i}}\right]$, initial history-dependent strategy *g* is distributed uniformly within the range of *g* ∈ [0, 1], starting price *P*(*t* = 0) is randomly chosen within the range of *P* ∈ [0, 20], each individual’s initial number of shares *k* = 0, each individual’s initial wealth *W*_*i*_(*t* = 0) = 0, each individual’s initial cash *C*_*i*_(*t* = 0) = 0.

The market efficiency is usually related to the predictability of the stock prices. In an efficient market, the stock price is unpredictable. In an inefficient market, the stock market is predictable. In order to examine whether the coexistence of different investment strategies affects the market efficiency or not, in Fig 7(a) and 7(b) we plot the predictability *H* of stock prices as a function of market impact *β* for different combinations of maximal risk tolerance *G*_*max*_ of reference point strategies and ratio *ξ* of individuals with reference point strategies [45].

Comparing the results in Fig 7(a) with the results in Fig 7(b), we find that an increase in the ratio *ξ* of individuals with reference point strategies has contradictory effects on the predictability *H* of stock prices. As *G*_*max*_ is relatively small, *G*_*max*_ = 100, which corresponds to the situation where the distribution of reference points is narrow, an increase in *ξ* leads to an overall increase in *H*. As *G*_*max*_ is relatively large, *G*_*max*_ = 5000, which corresponds to the situation where the distribution of reference points is wide, an increase in *ξ* leads to an overall decrease in *H*.

Such results indicate that the existence of individuals with history-dependent strategies leads to intermediate predictability of stock prices. The existence of individuals with homogeneous reference point strategies promotes the predictability of stock prices while the existence of individuals with heterogeneous reference point strategies suppresses the predictability of stock prices. The overall predictability of stock prices is determined by the characteristics of majority population. We can understand the simulation results as follows. The predictability of stock prices is related to the changing tendency of stock prices, which is determined by the difference in the numbers of individuals buying and selling the stocks. As the market impact is low or the risk tolerance is low, people’s frequently trading behavior leads to a large difference in the numbers of individuals buying and selling the stocks, which would make the moving tendency of stock prices become more predictable.

**Fig 8:**
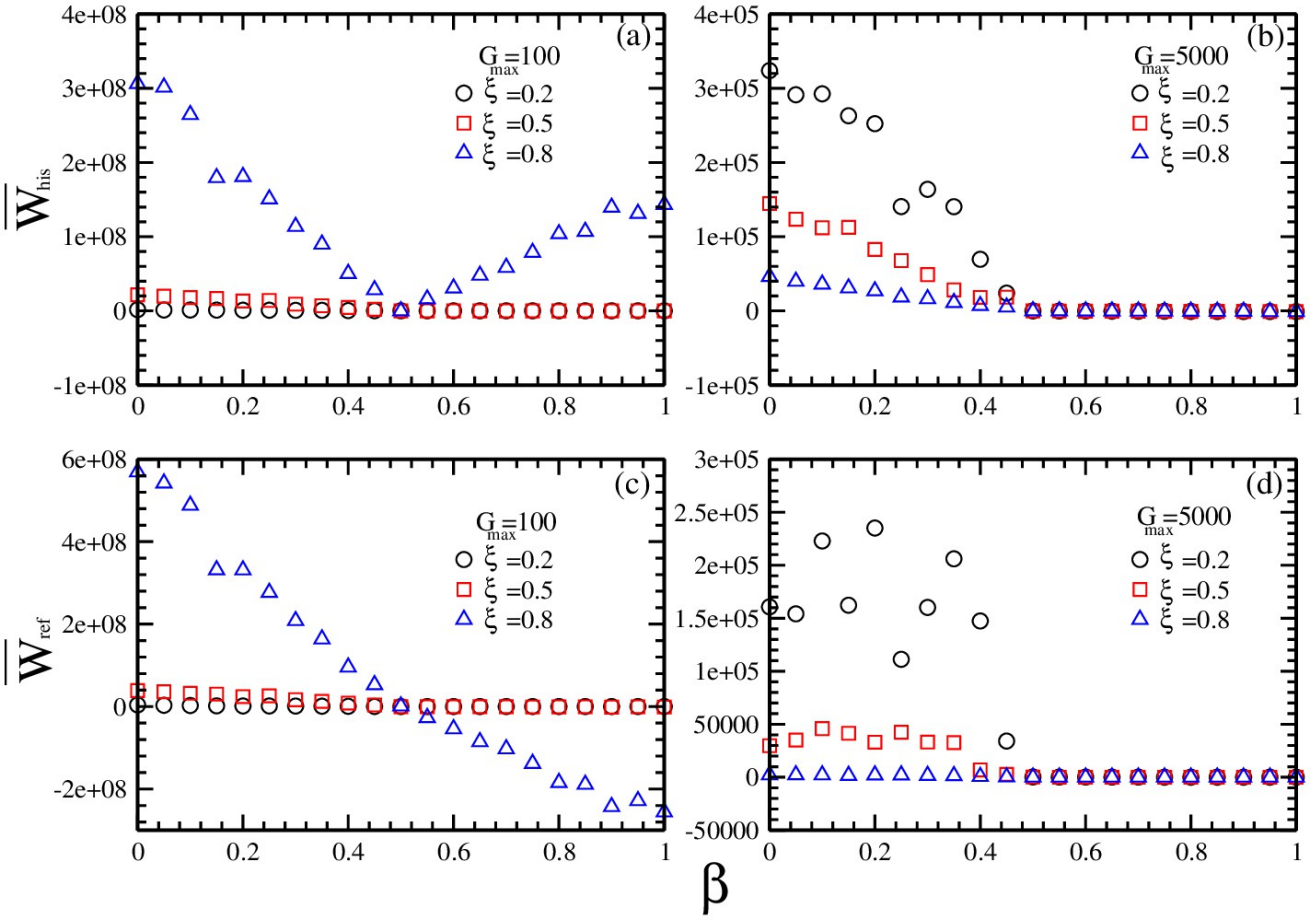
The average wealth ${\bar{W}}_{his}$ of individuals with history-dependent strategies and the average wealth ${\bar{W}}_{ref}$ of individuals with reference point strategies as a function of market impact *β* for different combinations of maximal risk tolerance *G*_*max*_ of reference point strategies and the ratio *ξ* of individuals with reference point strategies. (a) ${\bar{W}}_{his}$ for *G*_*max*_ = 100 and *ξ* = 0.2 (circles), 0.5 (squares), 0.8 (triangles); (b) ${\bar{W}}_{his}$ for *G*_*max*_ = 5000 and *ξ* = 0.2 (circles), 0.5 (squares), 0.8 (triangles); (c) ${\bar{W}}_{ref}$ for *G*_*max*_ = 100 and *ξ* = 0.2 (circles), 0.5 (squares), 0.8 (triangles); (d) ${\bar{W}}_{ref}$ for *G*_*max*_ = 5000 and *ξ* = 0.2 (circles), 0.5 (squares), 0.8 (triangles). Other parameters are: the population size *N* = 5001, the memory size *m* = 3 and *m*′ = 10, the updating threshold of history-dependent strategies *S*_*th*_ = 0, the maximum drift of history-dependent strategies *ε* = 0.1, the liquidity $\lambda =\frac{N}{10}$, the constant $\alpha =\frac{10}{N}$, minimum and maximum shares holding *k*_*min*_ = -1, *k*_*max*_ = 1. The initial conditions are: an individual’s risk tolerance *G*_*i*_ is uniformly distributed within the range of *G*_*i*_ ∈ [0, *G*_*max*_], an individual’s initial reference point $p_{i}^{ref}$ is uniformly distributed within the range of $p_{i}^{ref}\in \left[\bar{P}e^{-\alpha G_{i}},\bar{P}e^{\alpha G_{i}}\right]$, initial history-dependent strategy *g* is distributed uniformly within the range of *g* ∈ [0, 1], starting price *P*(*t* = 0) is randomly chosen within the range of *P* ∈ [0, 20], each individual’s initial number of shares *k* = 0, each individual’s initial wealth *W*_*i*_(*t* = 0) = 0, each individual’s initial cash *C*_*i*_(*t* = 0) = 0.

In order to find a competitive strategy in the investment, in Fig 8 we plot the average wealth of the individuals with history-dependent strategies and reference point strategies as a function of market impact *β* for different combinations of maximal risk tolerance *G*_*max*_ of reference point strategies and ratio *ξ* of individuals with reference point strategies.

Fig 8(a) and 8(c) show that, for *G*_*max*_ = 100, within the range of 0 ≤ *β* < 0.5, an increase in the ratio of the individuals with reference point strategies leads to an increase in the average wealth of the individuals with history-dependent strategies and the average wealth of the individuals with reference point strategies. Within the range of 0.5 < *β* ≤ 1, an increase in the ratio of the individuals with reference point strategies leads to an increase in the average wealth of the individuals with history-dependent strategies and a decrease in the average wealth of the individuals with reference point strategies.

Comparing ${\bar{W}}_{his}$ in Fig 8(a) with ${\bar{W}}_{ref}$ in Fig 8(c), we find that, for *G*_*max*_ = 100, within the range of 0 ≤ *β* < 0.5, ${\bar{W}}_{ref}$ is greater than ${\bar{W}}_{his}$. Within the range of 0.5 < *β* ≤ 1, ${\bar{W}}_{his}$ is greater than ${\bar{W}}_{ref}$. Comparing the results in Fig 7(a) with the results in Fig 8(a), we observe that the highest average wealth in Fig 8(a), i.e. $\bar{W}∼10^{8}$ for *β* = 0 and *G*_*max*_ = 100, is related to the predictability of transaction price *P*^*tr*^ and the fluctuation *σ*_*P*_ of stock prices. For *β* = 0, *P*^*tr*^(*t*) = (1 − *β*) *P*(*t* − 1) + *βP*(*t*) = *P*(*t* − 1). An individual would do a deal at the former price *P*(*t* − 1) which has been known to all the individuals. *P*^*tr*^(*t*) is predictable. Depending upon his accurate prediction, an individual would buy low and sell high. Therefore, *W*_*i*_ = *C*_*i*_ + *k*_*i*_ *P*^*tr*^ = Σ(*P*^*sell*^ − *P*^*buy*^) + *k*_*i*_ *P*^*tr*^ > 0. For *G*_*max*_ = 100, the price fluctuation is large, which would lead to a large attainment at a buying-selling transaction. Therefore, *C*_*i*_ = *Σ*(*P*^*sell*^ − *P*^*buy*^) would be quite large and the average wealth $\bar{W}=\frac{\Sigma W_{i}}{N}$ would reach its highest level.

Fig 8(b) and 8(d) show that, for *G*_*max*_ = 5000, within the range of 0 ≤ *β* < 0.5, an increase in the ratio of the individuals with reference point strategies leads to a decrease in the average wealth of the individuals with history-dependent strategies and the average wealth of the individuals with reference point strategies. Within the range of 0.5 < *β* ≤ 1, an increase in the ratio of the individuals with reference point strategies leads to an increase in the average wealth of the individuals with history-dependent strategies and a decrease in the average wealth of the individuals with reference point strategies.

Comparing ${\bar{W}}_{his}$ in Fig 8(b) with ${\bar{W}}_{ref}$ in Fig 8(d), we find that, for *G*_*max*_ = 5000, within the range of 0 ≤ *β* < 0.5, ${\bar{W}}_{his}$ is greater than ${\bar{W}}_{ref}$. Within the range of 0.5 < *β* ≤ 1, ${\bar{W}}_{ref}$ is greater than ${\bar{W}}_{his}$.

Such results indicate that whether an investment strategy can become a competitive strategy is determined by the coupling of market impact and the distributions of different investment strategies. For low market impact, a narrower distribution is beneficial for attaining more. For high market impact, a broader distribution is beneficial for attaining more. It is quite possible that a competitive strategy in the market with low market impact becomes a failure strategy in the market with high market impact, which can be seen as another explanation for the principle of risk-return equilibrium: high risk and high return, low risk and low return.

## Theoretical analysis

### Relationship between market impact and predictability of transaction prices

In the present model, the evolutionary dynamics is greatly affected by the market impact *β*. In the following, we give an analysis on how the market impact affects the predictability of transaction prices and then the evolutionary dynamics.

The transaction price at time t is related to the prices at time t-1 and time t,

$$\begin{array}{c} P^{tr}\left(t\right)=\left(1-\beta \right)P\left(t-1\right)+\beta P\left(t\right). \end{array}$$ (11)

For *β* = 0, the buyers and the sellers finish their transactions with the latest historical price *P*(*t* − 1). For *β* = 1, the buyers and the sellers finish their transactions with the instant price *P*(*t*).

An individual’s investment behavior is related to his attainment in the transaction, which depends on whether he can accurately predict the price movement or not. If an individual expects an increase in the stock price, he is quite possible to make a buying decision. If an individual expects a decrease in the stock price, he is quite possible to make a selling decision. If he could make a prediction accurately, he would buy low and sell high, which means *P*^*sell*^ − *P*^*buy*^ > 0. Therefore, his accumulated wealth *W*_*i*_(*t*),

$$\begin{array}{c} W_{i}\left(t\right)=\Sigma \left(P^{sell}-P^{buy}\right)+k_{i}P^{tr}, \end{array}$$ (12)

increases.

In the present model, before the transaction is finished, all the individuals know the historical price *P*(*t* − 1). For *β* = 0, *P*^*tr*^ = *P*(*t* − 1). The transaction price is predictable. The individual who does a deal according to his prediction would earn more. Most of the people tend to adopt the same strategy and the crowded effect occurs. Comparing the simulation results in Fig 5(a) with the results in Fig 8(a), we observe that, for *β* = 0, as the individual strategies cluster around *g* ∼ 0.5_−_ or *g* ∼ 0.5_+_, the average wealth is quite high. Our theoretical analysis is in accordance with the simulation results.

In the present model, before the transaction is finished, no one knows the instant price *P*(*t*). For *β* = 1, *P*^*tr*^ = *P*(*t*). The transaction price is unpredictable. Both the individual who does a deal according to his prediction and the individual who does a deal rejecting his prediction could not earn more. Most of the people tend to adopt his unique strategy and the crowd-anticrowded effect occurs. Comparing the simulation results in Fig 5(d) with the results in Fig 8(b), we observe that, for *β* = 1, as half of the individuals cluster around *g* ∼ 0 and another half of the individuals cluster around *g* ∼ 1, the average wealth is quite low. Our theoretical analysis is in accordance with the simulation results.

### Coevolution of history-dependent strategies and reference point strategies

Firstly, we give an analysis on how the history-dependent strategies and the reference point strategies evolve independently [74–76].

The evolution of history-dependent strategies is closely related to the prediction of individual i. If he could predict the change of stock prices correctly, he would buy low and sell high. The strategy he adopts would have a higher score and be kept continuously. If he could not predict the change of stock prices correctly, he would buy high and sell low. The strategy he adopts would have a lower score and be thrown away by most of the people. Whether an individual can predict the change of price is closely related to the market impact *β*. For *β*<0.5, the history-dependent strategies will finally evolve to the state where all the individuals adopt *g* > 0.5 strategies or *g* < 0.5 strategies. Given an initial uniform distribution of *g* ∈ [0, 1], whether the *g* < 0.5 strategies or the *g* > 0.5 strategies will finally become a dominant strategy is determined by the occasional advantage of *g*. For example, facing the latest change of prices ↑↑↑, if the history-dependent prediction is ↓ and most of the individuals adopt the *g* > 0.5 strategies, more sellers than buyers will lead to a decrease in price. The individuals with *g* > 0.5 strategies are quite possible to keep their strategies and the individuals with *g* < 0.5 strategies are quite possible to update their strategies, which finally leads to the situation where all the individuals adopt *g* > 0.5 strategies. Facing the latest change of prices ↑↑↑, if the history-dependent prediction is ↓ and most of the individuals adopt the *g* < 0.5 strategies, more buyers than sellers will lead to an increase in price. The individuals with *g* < 0.5 strategies are quite possible to keep their strategies and the individuals with *g* > 0.5 strategies are quite possible to update their strategies, which finally leads to the situation where all the individuals adopt *g* < 0.5 strategies. Therefore, given an initial uniform distribution of *g* ∈ [0, 1], the majority game effect will finally lead to the *δ*-like distribution of history-dependent strategies. In Fig 5(a), we observe that, for *ξ* = 0, the distribution of history-dependent strategies is a *δ*-like distribution clustering around *g* ∼ 0.5_−_ or *g* ∼ 0.5_+_.

For *β* > 0.5, the history-dependent strategies will finally evolve to the state where half of the individuals adopt *g* > 0.5 strategies and another half of the individuals adopt *g* < 0.5 strategies. For example, facing the latest change of prices ↑↑↑, if the history-dependent prediction is ↓ and most of the individuals adopt the *g* > 0.5 strategies, more sellers than buyers will lead to a decrease in price. The individuals with *g* > 0.5 strategies are quite possible to update their strategies and the individuals with *g* < 0.5 strategies are quite possible to keep their strategies, which finally leads to the situation where the individuals with *g* > 0.5 strategies decrease and the individuals with *g* < 0.5 strategies increase. Facing the latest change of prices ↑↑↑, if the history-dependent prediction is ↓ and most of the individuals adopt the *g* < 0.5 strategies, more buyers than sellers will lead to an increase in price. The individuals with *g* < 0.5 strategies are quite possible to update their strategies and the individuals with *g* > 0.5 strategies are quite possible to keep their strategies, which finally leads to the situation where the individuals with *g* < 0.5 strategies decrease and the individuals with *g* > 0.5 strategies increase. Therefore, given an initial uniform distribution of *g* ∈ [0, 1], the minority game effect will finally lead to the U-shape distribution of history-dependent strategies. In Fig 5(c), we observe that, for *ξ* = 0, the distribution of history-dependent strategies is a U-shape distribution clustering around *g* ∼ 0 and *g* ∼ 1.

The evolution of reference point strategies is closely related to the maximal risk tolerance *G*_*max*_. For a small *G*_*max*_, the reference point strategies finally evolve to the state where nearly all the individuals adopt the strategy $p^{exp}∼\bar{P}$. For example, for a quite small *G*_*max*_ = 1 and $\alpha =\frac{1}{100}$, facing the latest average price $\bar{P}$, Only the individuals with $\bar{P}e^{-0.01}\leq p^{ref}\leq \bar{P}e^{0.01}$ keeps their strategies. The individuals with $p^{ref}>\bar{P}e^{\alpha G_{max}}=\bar{P}e^{0.01}∼1.01\bar{P}$ or $p^{ref}<\bar{P}e^{-\alpha G_{max}}=\bar{P}e^{-0.01}∼0.99\bar{P}$ strategies will update their strategies. The frequent change of *p*^*ref*^ finally makes the reference point strategies cluster around $p^{exp}∼\bar{P}$. Therefore, given a quite small *G*_*max*_, the reference point strategies finally evolve to a narrow distribution clustering around $p^{exp}∼\bar{P}$. In Fig 5(e), we observe that, for *ξ* = 1, the distribution of reference point strategies is a narrow distribution clustering around $\frac{P^{ref}}{P_{max}^{ref}}∼0.65$.

For a large *G*_*max*_, the reference point strategies will finally evolve to the state where nearly all the individuals adopt heterogeneous strategies scattering between $p^{ref}∼\bar{P}e^{-\alpha G_{max}}$ and $p^{ref}∼\bar{P}e^{\alpha G_{max}}$. For example, for a quite large *G*_*max*_ = 1000 and $\alpha =\frac{1}{100}$, facing the latest average price $\bar{P}$, the individuals with $\bar{P}e^{-10}\leq p^{ref}\leq \bar{P}e^{10}$ keeps their strategies. Only the individuals with $p^{ref}>\bar{P}e^{G_{max}}=\bar{P}e^{10}∼10^{4}\bar{P}$ or $p^{ref}<\bar{P}e^{-G_{max}}=\bar{P}e^{-10}∼10^{-4}\bar{P}$ strategies will update their strategies. Therefore, given a quite large *G*_*max*_, the individuals with reference point strategies not far away from the average price is quite possible to keep their initial strategies. In Fig 5(f), we observe that, for *ξ* = 1, the distribution of reference point strategies is a broad distribution scattering between $\frac{P^{ref}}{P_{max}^{ref}}∼0$ and $\frac{P^{ref}}{P_{max}^{ref}}∼1$.

**Fig 9:**
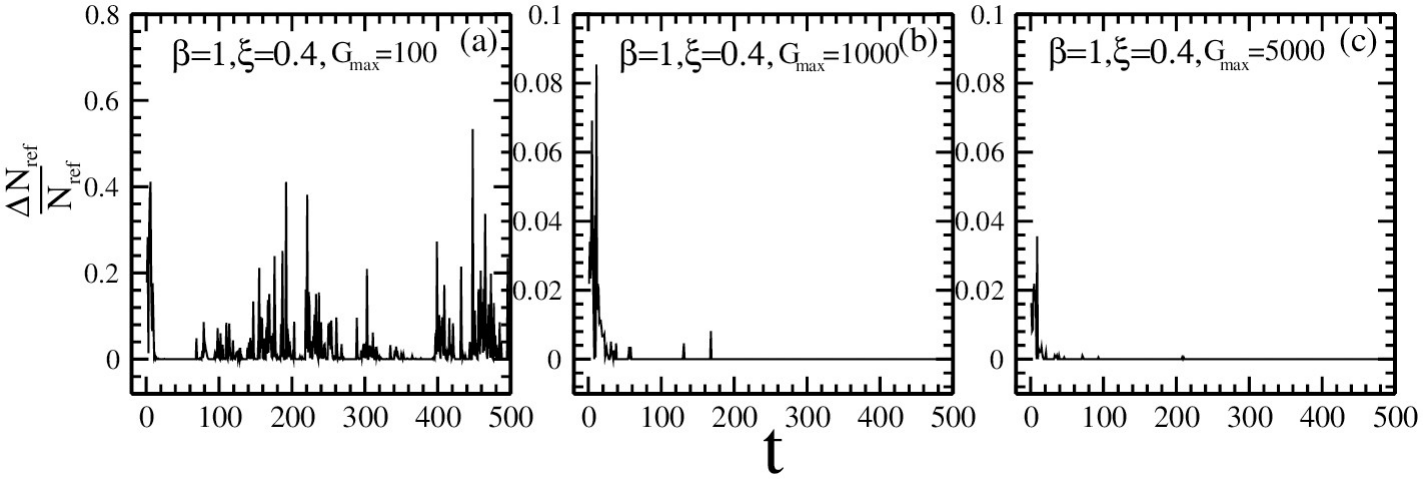
The ratio of individuals updating their reference point strategies dynamically for the maximal risk tolerance (a) *G*_*max*_ = 100; (b) *G*_*max*_ = 1000; (c) *G*_*max*_ = 5000. Other parameters are: the population size *N* = 5001, the market impact *β* = 1, the ratio of individuals with reference point strategies *ξ* = 0.4, the memory size *m* = 3 and *m*′ = 10, the updating threshold of history-dependent strategies *S*_*th*_ = 0, the maximum drift of history-dependent strategies *ε* = 0.1, the liquidity $\lambda =\frac{N}{10}$, the constant $\alpha =\frac{10}{N}$, minimum and maximum shares holding *k*_*min*_ = -1, *k*_*max*_ = 1. The initial conditions are: an individual’s risk tolerance *G*_*i*_ is uniformly distributed within the range of *G*_*i*_ ∈ [0, *G*_*max*_], an individual’s initial reference point $p_{i}^{ref}$ is uniformly distributed within the range of $p_{i}^{ref}\in \left[\bar{P}e^{-\alpha G_{i}},\bar{P}e^{\alpha G_{i}}\right]$, initial history-dependent strategy *g* is distributed uniformly within the range of *g* ∈ [0, 1], starting price *P*(*t* = 0) is randomly chosen within the range of *P* ∈ [0, 20], each individual’s initial number of shares *k* = 0, each individual’s initial wealth *W*_*i*_(*t* = 0) = 0, each individual’s initial cash *C*_*i*_(*t* = 0) = 0.

As a comparison, in Fig 9, we have plotted the ratio of the individuals updating their reference point strategies dynamically. We can observe that, as the maximal risk tolerance changes from *G*_*max*_ = 100 to *G*_*max*_ = 5000, the ratio of the individuals updating their reference point strategies dynamically reduces, which is in accordance with our theoretical analysis.

Secondly, we analyze how the existence of reference point strategies with a small *G*_*max*_ affects the evolution of history-dependent strategies.

For *β*<0.5, the majority-win effect dominates. The existence of individuals with reference point strategies leads to the situation where the history-dependent strategies will finally evolve to the state where all the individuals adopt *g* > 0.5 strategies. For example, facing the latest change of prices ↑↑↑, if the history-dependent prediction is ↓ and most of the individuals with history-dependent strategies adopt the *g* > 0.5 strategies, $\Delta N_{sell}^{his}=N_{sell}^{his}-N_{buy}^{his}>0$. At the same time, for the individuals with reference point strategies, a continuous rise in the prices, ↑↑↑, is quite possible to lead to more sellers than buyers, $\Delta N_{sell}^{ref}=N_{sell}^{ref}-N_{buy}^{ref}>0$. More seller than buyers, $\Delta N_{sell}=\Delta N_{sell}^{his}+\Delta N_{sell}^{ref}>0$, will lead to a decrease in price. The individuals with *g* > 0.5 strategies gain more and keep their strategies. Facing the latest change of prices ↑↑↑, if the history-dependent prediction is ↓ and most of the individuals with history-dependent strategies adopt the *g* < 0.5 strategies, $\Delta N_{sell}^{his}=N_{sell}^{his}-N_{buy}^{his}<0$. At the same time, for the individuals with reference point strategies, a continuous rise in the prices, ↑↑↑, is quite possible to lead to more sellers than buyers, $\Delta N_{sell}^{ref}=N_{sell}^{ref}-N_{buy}^{ref}>0$. For a large *ξ*, more seller than buyers, $\Delta N_{sell}=\Delta N_{sell}^{his}+\Delta N_{sell}^{ref}>0$, leads to a decrease in price. The individuals with *g* < 0.5 strategies lose more and update their strategies. Therefore, the existence of individuals with reference point strategies leads to the situation where the history-dependent strategies will finally evolve to the state where all the individuals adopt *g* > 0.5 strategies. Comparing the simulation results for *ξ* = 0 with the results for *ξ* > 0 in Fig 5(a), we observe that, an increase in the ratio of the individuals with reference point strategies makes the distribution of history-dependent strategies changes from a *δ*-like distribution clustering around *g* ∼ 0.5_−_ or *g* ∼ 0.5_+_ to a *δ*-like distribution clustering around *g* ∼ 0.5_+_, which is in accordance with our theoretical analysis.

For *β*>0.5, the minority-win effect dominates. The existence of individuals with reference point strategies leads to the situation where the history-dependent strategies will finally evolve to the state where all the individuals adopt *g* < 0.5 strategies. For example, facing the latest change of prices ↑↑↑, if the history-dependent prediction is ↓ and most of the individuals with history-dependent strategies adopt the *g* > 0.5 strategies, $\Delta N_{sell}^{his}=N_{sell}^{his}-N_{buy}^{his}>0$. At the same time, for the individuals with reference point strategies, a continuous rise in the prices, ↑↑↑, is quite possible to lead to more sellers than buyers, $\Delta N_{sell}^{ref}=N_{sell}^{ref}-N_{buy}^{ref}>0$. For a large *ξ*, more seller than buyers, $\Delta N_{sell}=\Delta N_{sell}^{his}+\Delta N_{sell}^{ref}>0$, will lead to a decrease in price. The individuals with *g* > 0.5 strategies lose more and update their strategies. Facing the latest change of prices ↑↑↑, if the history-dependent prediction is ↓ and most of the individuals with history-dependent strategies adopt the *g* < 0.5 strategies, $\Delta N_{sell}^{his}=N_{sell}^{his}-N_{buy}^{his}<0$. At the same time, for the individuals with reference point strategies, a continuous rise in the prices, ↑↑↑, is quite possible to lead to more sellers than buyers, $\Delta N_{sell}^{ref}=N_{sell}^{ref}-N_{buy}^{ref}>0$. For a large *ξ*, more seller than buyers, $\Delta N_{sell}=\Delta N_{sell}^{his}+\Delta N_{sell}^{ref}>0$, will lead to a decrease in price. The individuals with *g* < 0.5 strategies gain more and keep their strategies. Therefore, the existence of individuals with reference point strategies leads to the situation where the history-dependent strategies finally evolve to the state where all the individuals adopt *g* < 0.5 strategies. Comparing the simulation results for *ξ* = 0 with the results for *ξ* > 0 in Fig 5(c), we observe that, an increase in the ratio of the individuals with reference point strategies makes the distribution of history-dependent strategies changes from a U-like distribution clustering around *g* ∼ 0 and *g* ∼ 0 to a *δ*-like distribution clustering around *g* ∼ 0, which is in accordance with our theoretical analysis.

Fig 9 shows that, the existence of reference point strategies with a large *G*_*max*_ leads to $\Delta N_{buy}^{ref}∼0$ or $\Delta N_{sell}^{ref}∼0$. Therefore, the evolution of history-dependent strategies will not be affected by the existence of reference point strategies.

The above analysis indicates that the distribution of history-dependent strategies would be affected by the existence of the reference point strategies with a small *G*_*max*_ and keep unchanged in other cases, which is in accordance with the simulation results in Fig 5.

### Relationship between price fluctuations and strategy distributions

The price fluctuation is determined by the difference between the number of individuals buying and selling the stocks,

$$\begin{array}{c} lnP\left(t\right)-lnP\left(t-1\right)=\frac{N_{buy}-N_{sell}}{\lambda }. \end{array}$$ (13)

Suppose Δ*N* = ∣*N*_*buy*_ − *N*_*sell*_∣, Δ*N* is closely related to the combination of the distributions of history-dependent strategies and reference point strategies. For a *δ*–like distribution of history-dependent strategies coupled with a narrow distribution of reference point strategies, Δ*N* will reach its maximum value. For a *U*–like distribution of history-dependent strategies coupled with a broad distribution of reference point strategies, Δ*N* will reach its minimum value.

For a *δ*–like distribution of history-dependent strategies, the difference between the numbers of individuals buying and selling the stocks is satisfied with the condition ∣*g*_*th*_ − 0.5∣*N* ≤ Δ*N*_*his*_ ≤ *N*, in which *g*_*th*_ is the maximum value *g*_*max*_ for a *δ*–like distribution clustering around *g* < 0.5 and the minimum value *g*_*min*_ for a *δ*–like distribution clustering around *g* > 0.5. Comparing the simulation results in Fig 5 with the results in Fig 4, we observe that, within the range of *β* < 0.5 and *G*_*max*_ = 100, the *δ*–like distribution of history-dependent strategies corresponds to a large value of *σ*_*P*_.

For a *U*–like distribution of history-dependent strategies, the difference between the numbers of individuals buying and selling the stocks is satisfied with the condition Δ*N*_*his*_ ∼ 0. Comparing the simulation results in Fig 5 with the results in Fig 4, we observe that, within the range of *β* > 0.5 and *G*_*max*_ = 5000, the *U*–like distribution of history-dependent strategies corresponds to a small value of *σ*_*P*_.

For a narrow distribution of reference point strategies clustering around $\bar{P}$, the difference between the numbers of individuals buying and selling the stocks is satisfied with the equation $\Delta N_{ref}∼N\int f\left(\bar{P},\sigma_{p^{ref}}\right)dP$, in which $f(\bar{P},\sigma_{p^{ref}})$ is the probability density function. Comparing the simulation results in Fig 5 with the results in Fig 4, we observe that, within the range of *β* < 0.5 and *G*_*max*_ = 100, a narrow distribution of reference point strategies corresponds to a large value of *σ*_*P*_.

For a broad distribution of reference point strategies around $\bar{P}$, the difference between the numbers of individuals buying and selling the stocks is satisfied with the equation $\Delta N_{ref}∼\frac{N\Delta P}{\bar{P}e^{\alpha G_{max}}+\bar{P}e^{-\alpha G_{max}}}$. Comparing the simulation results in Fig 5 with the results in Fig 4, we observe that, within the range of *β* > 0.5 and *G*_*max*_ = 5000, a broad distribution of reference point strategies corresponds to a small value of *σ*_*P*_.

For a *δ*–like distribution of history-dependent strategies coupled with a narrow distribution of reference point strategies, Δ*N* becomes $|g_{th}-0.5|N\left(1-\psi \right)+\psi N\int f\left(\bar{P},\sigma_{p^{ref}}\right)dP\leq \Delta N_{his}+\Delta N_{ref}\leq N\left(1-\psi \right)+\psi N\int f\left(\bar{P},\sigma_{p^{ref}}\right)dP$. Comparing the simulation results in Fig 5 with the results in Fig 4, we observe that, within the range of *β* < 0.5 and *G*_*max*_ = 100, a *δ*–like distribution of history-dependent strategies coupled with a narrow distribution of reference point strategies leads to an overall increase in *σ*_*P*_.

For a *U*–shape distribution of history-dependent strategies coupled with a broad distribution of reference point strategies, Δ*N* becomes $0\leq \Delta N_{his}+\Delta N_{ref}∼\frac{\psi N\Delta P}{\bar{P}e^{\alpha G_{max}}+\bar{P}e^{-\alpha G_{max}}}$. For a large *G*_*max*_ ∼ *N*, we find that $N\left(1-\psi \right)+\psi N\int f\left(\bar{P},\sigma_{p^{ref}}\right)dP>>\frac{\psi N\Delta P}{\bar{P}e^{\alpha G_{max}}+\bar{P}e^{-\alpha G_{max}}}$. Comparing the simulation results in Fig 5 with the results in Fig 4, we observe that, within the range of *β* > 0.5 and *G*_*max*_ = 5000, a *U*–shape distribution of history-dependent strategies coupled with a broad distribution of reference point strategies leads to an overall decrease in *σ*_*P*_.

The above analysis implies that a *δ*–like distribution of history-dependent strategies coupled with a narrow distribution of reference point strategies should lead to the largest price fluctuation and a *U*–shape distribution of history-dependent strategies coupled with a broad distribution of reference point strategies should lead to the smallest price fluctuation. Therefore, the theoretical analysis is in accordance with the simulation results in Figs 4 and 5.
